# Immunosuppressive therapy for Vogt-Koyanagi-Harada disease: a retrospective study and review of literature

**DOI:** 10.1186/s12348-023-00333-6

**Published:** 2023-05-19

**Authors:** Najiha Rahman, Jose Carlo M Artiaga, Konstantinos Bouras, Joshua Luis, Angela Rees, Mark Westcott

**Affiliations:** 1 Uveitis Service, Moorfields Eye Hospital, 162 City Road, London, EC1V 2PD UK; 2 Institute of Ophthalmology, University College London (UCL), London, UK; 3 William Harvey Research Institute, Queen Mary University of London, London, UK

**Keywords:** Uveitis, Vogt-Koyanagi-Harada, Immunosuppressive, Treatment, Mycophenolate mofetil, Adalimumab, Azathioprine, Ciclosporin

## Abstract

**Background:**

Vogt-Koyanagi-Harada (VKH) disease is an idiopathic autoimmune disease which targets melanin-containing tissues such as the uvea, meninges, ear and skin. This typically presents in the eye with acute findings of granulomatous anterior uveitis, diffuse choroidal thickening, multiple focal areas of sub-retinal fluid and, in severe cases, optic nerve involvement with bullous serous retinal detachment can occur.

Early initiation of treatment has been advocated to prevent progression to the chronic stage of the disease, which can result to a sunset glow fundus with devastatingly poor visual outcome. Treatment is usually initiated with corticosteroids followed by an early introduction of immunosuppressive treatment (IMT) to achieve immediate response after disease presentation, although the choice of IMT for VKH can vary.

**Main Findings:**

We conducted a retrospective case-series to investigate the management trend of treating VKH over a 20-year period. Twenty-six patients were included and we found a shift from steroid monotherapy to combined IMT/low-dose steroid for the management of acute initial-onset of VKH in the last 10 years. Our average time from diagnosis to initiation of IMT was 2.1 months. 81% (21 of 26 patients) of our patients treated with combined IMT/steroid were able to achieve disease stability with significant good visual outcome at 24 months (Median VA_pre-IMT_ = 0.3 Logmar vs VA_post-IMT_ = 0.0 Logmar, p = 0.0001). MMF monotherapy was the most common IMT used and it was well-tolerated by our patients. Even so, 50% of our patients who were treated with MMF did not achieve disease control.

We then performed a literature review to identify any IMT which could be superior in the treatment of VKH. We also share our experience (where applicable) on the various treatment options found from the literature review.

**Short conclusion:**

Our study found that patients with VKH who were treated with combined IMT/low-dose steroids achieved significantly better visual improvement at 24 months compared to steroid monotherapy. We frequently chose MMF and this appears to be well tolerated by our patients. Since its introduction, anti-TNF agents are increasingly becoming a popular choice of treatment for VKH as these have been shown to be safe and effective. However, more data is required to provide evidence that anti-TNF agents can be used as first-line treatment and as monotherapy.

## Introduction

The description of Vogt-Koyanagi-Harada (VKH) disease follows a series of historical clinical discovery [1]. After a series of initial common findings of non-traumatic uveitis and complicated retinal detachment associated with poliosis, vitiligo, alopecia and dysacousia by Vogt, Koyanagi and Harada between 1906 and 1926, it led to the eventual eponym “Vogt-Koyanagi-Harada” (VKH) disease in 1955 [2, 3], which represents the clinical manifestations of an evolutionary disease process [4] connecting the uveal tract, the meninges, and the auditory and integumentary system.

The management of VKH has also evolved over time. For specific uveitis conditions such as VKH, immediate use of immunosuppressive treatment (IMT) within a therapeutic window is warranted to improve disease outcome. Herbort et al. recommended to initiate dual systemic steroid with IMT within 2 to 3 weeks of acute-onset VKH in order to achieve an improved outcome and preventing chronic evolution to “sunset glow fundus” [5]. Various management regimes have been described for VKH disease including local, oral, intravenous corticosteroids, ciclosporine, azathioprine and mycophenolate mofetil (MMF)[6–9]. Our colleagues in Saudi Arabia have found that MMF is particularly effective in VKH as it leads to significantly improved vision with reduced recurrence of inflammation and chronic complications [10]. More recently, the development of anti-TNF biologics has contributed as a potential game-changer for the treatment of other non-infectious uveitis conditions, however its reported efficacy for patients with VKH is based on small sample studies [1] and cases of refractory VKH [11].

### Our experience in the management of VKH

We conducted a retrospective case-series that gave us an overview of the management trend for VKH over a 20-year period in a unit that provides tertiary service for an ethnically diverse population in Central London. We have found a shift from steroid monotherapy to combined IMT/low-dose steroid for the management of acute initial-onset VKH in the last 10 years (Table 1). Our case-series of 26 consecutive patients diagnosed with VKH found 81% of patients were able to achieve disease stability with significant good visual outcome at 24 months, with a group median visual acuity (VA) better than baseline (Median VA_pre-IMT_ = 0.3 Logmar vs VA_post-IMT_ = 0.0 Logmar, *p* = 0.0001). IMT appears to be a well-tolerated long-term treatment for patients with VKH. In this case-series, although both treatment strategies resulted in good visual outcome, combined IMT and steroid provided a better visual improvement and greater probability of disease stability at 24 months compared to steroids alone (Table 2). Our average time from diagnosis to initiation of IMT was 2.1 months (Table 1). MMF monotherapy was the most common IMT used (68%, 13/19 patients treated with IMT).

**Table 1:** Patient demographics in our study (*n* = 26)

**Sex (%)**	
**Male**	**8 (31%)**
**Female**	**18 (69%)**
**Median Age**	**35 (Range 8–60)**
**Ethnicity (%)**	
**Asian**	**10 (38%)**
**African/Caribbean**	**3 (12%)**
**Caucasian**	**3 (12%)**
**Other**	**10 (38%)**
**Treatment Received**	
**Prednisolone only**	**7 (27%)**
**Prednisolone and IMT combination**	**19 (73%)**
**Mycophenolate mofetil (MMF)**	**13**
**Azathioprine (Aza)**	**3**
**Cyclosporin A (CsA)**	**3**
**Anti-TNF + other IMT**	**6**
**Average time from steroid initiation to IMT initiation**	**2.1 months**
**Average time to steroid-sparing effect of IMT (Prednisolone ≤ 7.5 mg)**	**5 months**

**Fig. 1:**
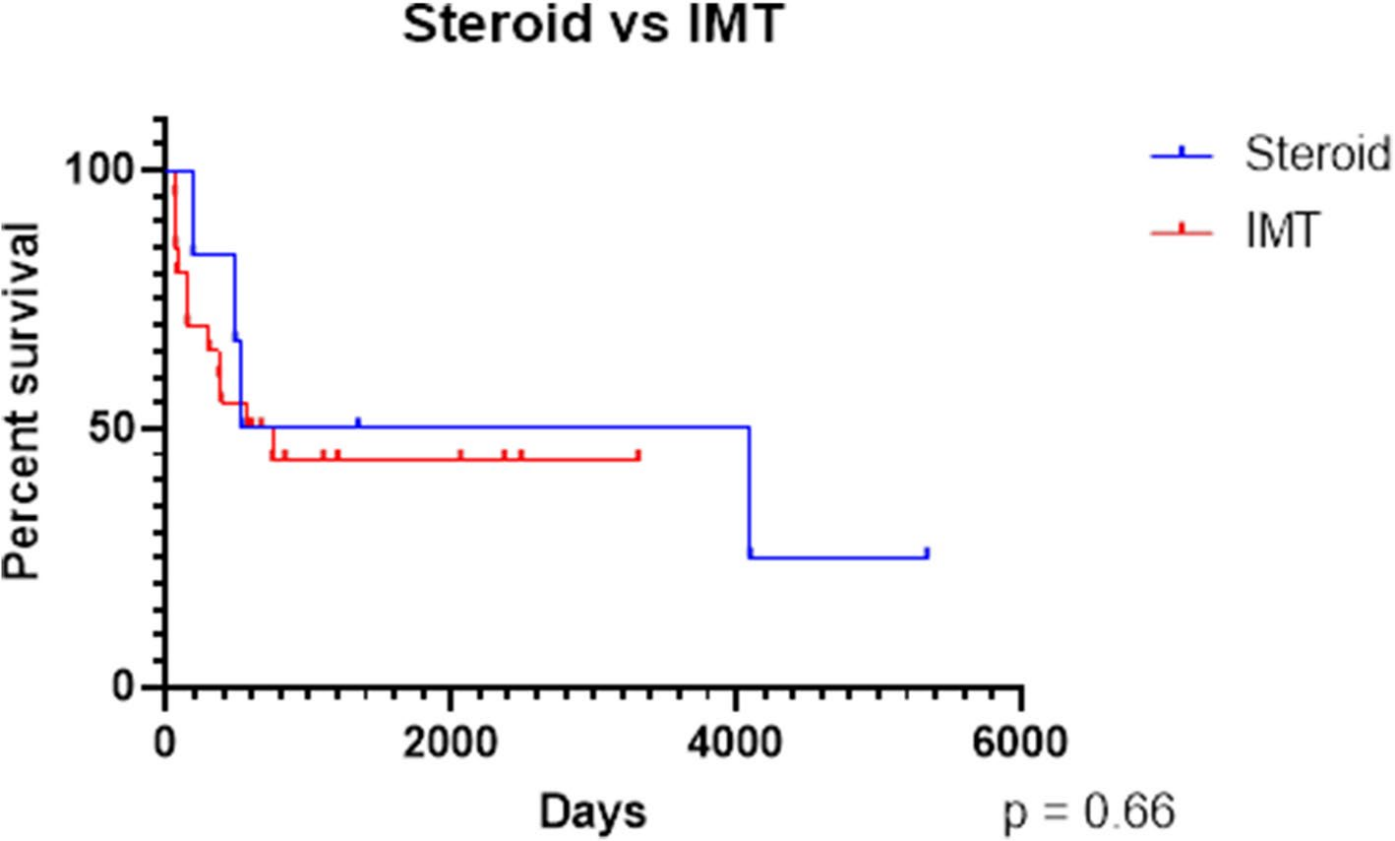
Kaplan–Meier survival analysis showing the time from presentation to time of reactivation in the steroid only and combination therapy groups

We also performed a survival analysis to measure the time from presentation to time of reactivation in the two groups respectively (Fig. 1).

**Table 2:** Visual outcomes following treatment

	Pre-treatment median VA	Median VA at 24 months	*p*-value
**Steroid only**	0.5 (Snellen = 6/18)	0.3 (Snellen = 6/12)	0.09
**Steroid + IMT**	0.3 (Snellen = 6/12)	0 (Snellen = 6/6)	< 0.001

Both groups showed similar levels of reduced activity to 50% by 2 years. Patients with IMT appeared to reach a plateau level of inactivity earlier than those on steroid monotherapy. Even so, the probability of active VKH with IMT is reduced to 45% at around 4 years of treatment, which then plateaued up to 9 years, whereas patients treated with corticosteroids had 25% probability of reactivation with longer duration of treatment up to 5–6 years. Duration of follow-up was shorter for the IMT group than the steroid group. There was no statistical difference in survival between the groups. We suspect that this is due to the small sample size.

The differences in the efficacy of VKH treatment among our group of patients may be a reflection of the clinicians’ preference and/or the patient’s disease course. This led us to perform a literature review with the primary aim to identify any potential IMT(s) with good efficacy for the treatment of VKH and also to provide an update on the different treatment options available in managing this disease-specific condition.

## Methodology

The review of literature was carried out in PubMed using the terms “vogt-koyanagi-harada” and individual immunomodulatory agents, such as “mycophenolate mofetil”, “azathioprine”, “methotrexate”, “cyclosporine”, “tacrolimus”, “adalimumab”, and “rituximab”. Boolean operators were used to include only relevant studies. Only articles with English abstracts were reviewed for inclusion in this review. Citation searching was also done to further include relevant studies. Retrieved studies were reviewed according to level of evidence (clinical trials, clinical studies, case series, case reports) and discussed accordingly.

## Literature review of VKH treatment

### Corticosteroids

Historically, corticosteroids were the mainstay of treatment for VKH [12]. In the early 1990s, studies showed that about 60% of VKH patients were able to attain good visual outcome better than 20/30 following high-dose corticosteroids [12–14] and the initial high-dose can be as high as 80–200 mg per day. In one of the earlier non-randomized studies on steroid therapy, Sasamoto et al. compared 18 patients on pulse-dose (3 days of intravenous methylprednisolone (IVMP) followed by gradual taper of oral steroids), 20 patients on high-dose corticosteroid starting at 200 mg Prednisolone, 2 patients on conventional steroid dose and 7 patients on no corticosteroids for the treatment of VKH [15]. They found that patients who had pulse- and high-dose corticosteroid treatment had significantly reduced AC inflammation and improved VA compared to those without treatment. One other study found that the highest risk of ocular inflammation occur during the first 6 months of the disease and this was associated with too rapid tapering of the oral steroids [13]. Rubsamen and Gass also identified three predictive factors for poor visual outcome in VKH, which included older age at presentation, chronic ocular inflammation and subretinal choroidal neovascular membrane, for which they recommended an early, aggressive use of systemic corticosteroids in patients with Vogt-Koyanagi-Harada syndrome and a gradual tapering of drug dosage for 6 months after presentation. However, our better understanding of the disease evolution over the last two decades has proven that it is difficult to sustain oral corticosteroids monotherapy use for a longer period of time and in fact, it may be insufficient to prevent chronic evolution [16].

In our experience, patients treated with steroid monotherapy (7/26) 71% achieved disease inactivity at 24 months and 2 patients developed cataract and glaucoma which are known ocular side effects of corticosteroid use. We used the conventional steroid dose for the initial treatment of VKH, i.e. 1 mg per kg of daily Prednisolone, and this group of patients attained significant final visual outcome of better than 6/7.5. In comparison, in our patients treated with IMT, a higher proportion (81%) achieved disease stability. The patients’ VA also improved, with group median VA significantly better at 24 months compared to baseline (Median VA_pre-IMT_ = 0.3 Logmar vs VA_post-IMT_ = 0.0 Logmar, p = 0.0001). This is in line with a study by Paredes et al. which suggested that IMT as first-line therapy for VKH was associated with a superior visual outcome compared to steroids either as monotherapy or with delayed addition of IMT. Early introduction of IMT is therefore recommended, not only to achieve better disease inflammatory control but also to prevent significant systemic side effects related to prolonged use of oral corticosteroids.

### Immunosuppressive treatment for VKH

#### Randomized controlled trial (RCT)

There are limited clinical trials that have compared the different IMT in the treatment of VKH. The First-line Antimetabolites as Steroid-Sparing Treatment (FAST) uveitis trial randomized 216 patients with non-infectious uveitis into methotrexate (MTX) and mycophenolate mofetil (MMF) groups [17]. Of these, 93 patients, or 43%, were diagnosed with VKH. The study concluded that the use of mycophenolate was not superior to methotrexate in terms of controlling inflammation in non-infectious uveitis. Although treatment success among posterior uveitis and panuveitis appeared to have been better achieved with methotrexate, the authors encouraged more studies into anatomical subtypes of uveitis. Forty-three patients diagnosed with VKH from the FAST trial were included in a sub-analysis conducted by Shen et al. which compared 1 g mycophenolate mofetil (MMF) twice daily and 25 mg methotrexate (MTX) once weekly. They found no statistically significant advantage in terms of steroid-sparing inflammatory control, although they did find that MTX was 2.5 times more likely to achieve control of inflammation in acute VKH than MMF [18].

Another randomized controlled trial (RCT) compared the combination of azathioprine (AZA) and prednisone to cyclosporine A (CsA) and prednisone in 44 VKH patients with chronic inflammation despite steroid treatment. Patients in the AZA group required a significantly higher average and total cumulative prednisone dose to achieve control [9]. They also concluded that CsA seems to be a better glucocorticoid-sparing agent for active VKH compared to AZA.

#### Cyclosporine A (CsA)

The immunologic process driving VKH is believed to be at least in part a T-cell mediated autoimmunity against antigenic components of uveal melanocytes [19]. Cyclosporine A (or ciclosporin) is a potent calcineurin inhibitor which inhibits the production of cytokines via regulation of T-cell activation [19]. This is usually given at a dose of 3–5 mg/kg/day and monitored for known side effects such as hypertension and deranged kidney function. Treatment with a combination of steroids and CsA has been found to decrease pro-inflammatory IFN-y and IL-17 and upregulate the anti-inflammatory IL-37 in the peripheral blood cells of VKH patients [20, 21]. In the same case series of Liu et al., all 8 patients with chronic VKH had no active inflammation and improved vision after treatment with a combination of steroids and CsA [21]. A recent article by Ono et al. showed that the same combination of CsA with corticosteroid therapy is non-inferior to corticosteroids alone in terms of risk of recurrence or worsening [22]. The risk of recurrence or worsening, sunset glow fundus rate, and cataract rate were all lower in the combination group than the corticosteroid group, suggesting an advantage to the combined regimen [22]. CsA was the drug of choice for steroid-resistant VKH in large retrospective cohort studies in Japan, Tunisia and China [4, 23, 24]. The use of CsA was also found to decrease the rate of recurrence of inflammation [23]. A study by Tagirasa et al. found CsA to effectively switch off inflammation among multi-drug resistant VKH previously treated with azathioprine [25]. A small study in Japan showed the effectiveness of low-dose CsA in steroid-resistant cases [26]. Numerous case reports have been published to demonstrate its efficacy in both adult and pediatric populations. However, our experience with CsA can be described as somewhat cautious, mainly due to its cardiovascular and renal side effects. We tend to use CsA as a temporary and short-term measure as we take the advantage of its rapid effect for inflammatory control.

#### Tacrolimus (Tac)

Tacrolimus is also a calcineurin inhibitor which acts by downregulating the cytokine interleukin-2, which then inhibits the actions of CD4 + T-cells. There have been only limited reports on the use of Tac specifically for VKH, and no RCT. An early clinical trial by Mochizuki et al. examined the effect of different doses of Tac on 53 patients with refractory uveitis (5 patients had VKH) [27]. A retrospective review by Luis et al. of 71 patients showed good effectiveness and safety of Tac in the treatment of noninfectious uveitis [28]. Of these, only 3 were diagnosed with VKH. In our retrospective review, we had two VKH patients treated with Tac as second-line following inefficacy of primary treatment. In both cases its use were short-lived due to side effects including reduced lymphocyte count and gastrointestinal disturbances.

#### Azathioprine (AZA)

Azathioprine is an immunosuppressant agent that acts through purine synthesis inhibition. In a retrospective case series by Kim et al., AZA was added to corticosteroids in the treatment of VKH. A corticosteroid-sparing effect was achieved in 86.5% of patients in the acute uveitic phase and in 90% of patients in the chronic recurrent phase of the disease [8]. The median time to achieve this effect was 3.5 months. AZA was the IMT of choice in 55% of patients being started on early IMT ≤ 3 months after disease onset in a retrospective study done in Korea. This study reported superior outcomes of early IMT versus conventional treatment in both visual and inflammatory outcomes of VKH management [29]. In a retrospective study conducted in India, episodes of VKH recurrence were primarily treated with a combination of steroids and AZA [30]. Complete resolution of inflammation with avoidance of sunset glow fundus was achieved in these 4 cases. In contrast, Lavezzo et al. reported persistence of subclinical inflammatory signs on imaging in VKH patients predominantly treated with azathioprine as first-line steroid-sparing agent in a cohort of 22 patients [31]. Bongomin et al. described a case of VKH in an Ugandan successfully treated with a combination of steroids and azathioprine [32]. AZA is also one of the safer immunosuppressants to take during pregnancy and was hence used successfully to supplement systemic and local steroids in a pregnant patient [33]. This patient was reportedly able to maintain excellent vision and good inflammatory control months after the delivery of pregnancy. AZA appeared to perform well among the 3 patients included in our case-series as they were able to achieve disease remission, including one patient who decided to stop all treatment at the early stages of pregnancy.

#### Mycophenolate mofetil (MMF)

Mycophenolate mofetil is a prodrug of mycophenolic acid which inhibits the production of B and T cells. In the FAST RCT sub-analysis, Shen et al. found that 53% of VKH patients treated with MMF achieved treatment success within the study period of 6 months [18]. However, this observation was not statistically significantly different when compared to methotrexate. El-Asrar and colleagues have published many studies on the efficacy of MMF in managing patients with VKH given the high prevalence of this condition in Saudi Arabia. In a non-randomized prospective study, they initially compared prospective outcomes of VKH patients given a primary treatment of combination MMF and oral steroids to a separate group receiving oral steroids alone among VKH patients. They found that the MMF group achieved better visual outcomes, reduced incidence of recurrence, and less complications than the steroid group [10]. Subsequently, a later prospective cohort study was able to demonstrate that MMF also prevented chronic recurrent granulomatous inflammation and development of sunset glow fundus [34]. Ninety-three percent of 76 eyes receiving MMF therapy achieved 20/20 VA with a median duration of 20.2 months of treatment. Approximately 60% of these patients were able to come off MMF without relapse of inflammation, while about 9% developed ocular complications related to VKH, i.e. 2 eyes developed glaucoma and 5 eyes developed cataract.

Other studies have also found good efficacy of MMF as treatment choice for VKH. In a 12-year retrospective study, MMF was used in 11% of patients and it was found to result in a significant visual improvement from baseline [35]. The use of MMF likewise was reported to be effective in atypical cases [36–38]. Our experience with MMF has been fair as the 5 out of 10 patients included in our study achieved disease remission and the medication was well tolerated by the patients. Fifty percent did not achieve disease control. We noted that 3 of the 10 patients developed anaemia with MMF. This is a reported adverse effect of MMF especially among transplant patients [39–41]. This is thought to be due to mycophenolic acid inhibition of inosine 5’-monophosphate dehydrogenase activity in erythroid cells as demonstrated in in vitro animal studies [42].

### Methotrexate (MTX)

Methotrexate is an anti-metabolite which acts by inhibiting dihydrofolate reductase and promoting T-cell apoptosis. The FAST sub-analysis identified that MTX was 2.5 times more likely to achieve steroid-sparing control of inflammation compared to MMF [18]. This finding was however not statistically significant, possibly because of the small number of patients with VKH in the FAST trial. A 17-year retrospective case series conducted in Colombia reported the use of MTX as adjuvant to steroids in 6 out of 25 VKH cases [43]. Four patients responded to MTX, while 2 experienced relapses and were shifted to other immunosuppressants. MTX has a known efficacy in childhood uveitis and we reviewed two case series that investigated the use of MTX among pediatric VKH patients [44, 45]. Kondo et al. reported good steroid-reducing effect and control of inflammation in all 3 cases of pediatric VKH [44]. Similarly, Soheilian et al. found that 6 out of 10 pediatric VKH patients who required a non-steroidal immunosuppressant achieved good inflammatory control with MTX [45]. One case was reported in literature wherein MTX was combined with infliximab for up to 10 years in a pediatric patient [46] with no relapsing inflammatory episodes and resolution of physical features of Cushing's syndrome were observed. In our series, none of the patients were treated with MTX.

#### Rituximab (RTX)

Rituximab is a fully humanized anti-CD20 antibody which acts by causing a depletion of pathogenic B-cells for up to 6 months. Although it has been approved by the US Food and Drug Administration for various indications, it has gained increasing acceptance in the treatment of ocular inflammatory conditions. Its efficacy in VKH has been described in only a few reports to date. In a retrospective study by Abu El-Asrar et al., 9 patients who failed with conventional immunosuppressive therapy achieved disease remission, significant visual improvement, and steroid-sparing effect following 3 rituximab infusions [47]. A retrospective series by Bolletta et al. included 5 patients who were refractory to conventional immunosuppressive therapy and reported significant visual improvement and reduction in sub-foveal choroidal thickness following at least 3 infusions of rituximab [48]. Dolz-Marco et al. first reported on the positive response of VKH to rituximab in a 41-year old female who was refractory to steroids, adalimumab, cyclosporine and methotrexate [49]. Long term remission with recovery of hearing loss was reported by Caso et al. [50]. A similar positive response was reported in a 10-year old girl who was recalcitrant to initial immunosuppressive therapy [51]. Of these cases, 2 were previously on adalimumab, suggesting that rituximab may be an option in cases not responding to anti-TNF alpha treatment.

### Adalimumab (Ada)

Adalimumab is a fully humanized IgG1 monoclonal antibody that neutralizes tumour necrosis factor (TNF)-alpha activity and induces apoptosis of TNF-expressing mononuclear cells. It is the only US Federal Drug Agency (US FDA) approved anti-TNF’s for uveitis which has been described in the treatment of VKH. At present, this agent is mostly used following failure of a combination of steroids and initial non-steroidal immunosuppressive therapy. Two landmark trials, VISUAL I and VISUAL II, examined the efficacy and safety of adalimumab in active (VISUAL I) and inactive (VISUAL II) intermediate, posterior, and panuveitis [52, 53]. VISUAL I reported lower risk of uveitic flare and visual impairment, but with more adverse events and serious side effects than placebo [52]. VISUAL II reported similar lower risk for flare up and visual impairment but with similar safety outcomes compared to the steroid-controlled placebo group [53]. Patients diagnosed with VKH comprised 12% and 22% of the two trials, respectively. In a retrospective study, Couto et al. examined the effect of adding Ada to the combination corticosteroids and conventional IMT (MTX, MMF and AZA) in 14 patients with VKH [54]. They found that the addition of Ada was able to reduce the combined mean corticosteroid dose of all patients, as well as the proportion of patients who are still on conventional IMT at 6 months. A Korean observational study by Park et al., which involved 3 VKH cases among 23 non-infectious uveitis cases, reported the efficacy of Ada in reducing anterior chamber cells, vitreous haze, and central macular thickness [55]. A larger, multi-centered study of 96 non-infectious uveitis (including VKH) patients reported good anti-inflammatory outcomes [56]. Subfoveal choroidal thickness, which may be a helpful indicator of treatment response in VKH, was effectively reduced with an Ada regimen in a series of 33 eyes with refractory non-infectious uveitis (42% with VKH) [57]. Three cases of VKH refractory to corticosteroids and either cyclosporine [58] or methotrexate [59] were shown to respond well after shifting treatment to Ada. Our Italian Paediatric Rheumatology colleagues with special interest in childhood uveitis reported a case of an 8-year-old boy treated for VKH using Ada as the primary steroid-sparing immunosuppressant following 3-day IVMP and this achieved good control of inflammation within 8 months [60].

Four other TNF alpha inhibitors are approved by the US FDA for other indications [61]. Literature describing the use of infliximab and certolizumab is limited to a few case reports. Infliximab is a chimaeric IgG anti-human monoclonal antibody has been reported to be effective in both adult and pediatric patients refractory to first-line IMT ([46, 62–64]. Infliximab has also been used as first-line IMT (with methotrexate) following high-dose steroids in 2 adult and 2 pediatric patients and was successful in achieving inflammatory control [65, 66]. Certolizumab pegol is a pegylated Fab fragment anti-TNF alpha drug. One patient with VKH was included in a retrospective multi-centre case series which reported that certolizumab is able to achieve or maintain control of eye inflammation during pregnancy in all study patients [67]. There were no studies describing the use of 2 other anti-TNF’s (etanercept and golimumab) for VKH.

### Systemic steroid-free therapy with Ada and IMT

A single centre large cohort study from China evaluated the use Ada and IMT alone without the use of systemic corticosteroid (“systemic glucocorticoid free”, or SGF) [68]. Thirty patients naïve to systemic therapy were recruited. Fifteen received conventional therapy with steroids and IMT with either MTX, MMF, or CSA, while the other 15 received SGF. They showed that the SGF therapy was safe, and well tolerated. In addition, the regimen achieved similar rates of efficacy in terms of control of anterior uveitis, vitritis, and normalizing central macular thickness, compared to the group treated with systemic steroids and IMT. The authors commented that the SGF regimen is an option in VKH patients, thereby avoiding the side effects of systemic steroids. The SGF regime is also attractive in those patients who have other co-morbidites that are exacerbated or contraindicated by the use of systemic steroids, for example diabetes.

## Discussion

Based on the results of our case-series and this literature review, we would recommend early introduction of steroid – sparing agents in treating VKH and MMF as the first-line treatment of choice in the management of VKH cases. Although well tolerated in terms of its commonly reported gastrointestinal side effects, it is also important to note that MMF can also cause anaemia. MMF has good efficacy as demonstrated by El-Asrar and colleagues in Saudi Arabia where VKH is more prevalent, hence they had good number of patients included in their studies, which is more reliable to indicate drug efficacy.

Although numerous studies in Japan and China where VKH is also prevalent showed CsA to be as efficacious, we exercise caution in using CsA due to its significant systemic side effects. Our tendency is to use CsA as a temporary measure in the acute phase of the disease in order to achieve a more immediate inflammatory control before switching to MMF as the long-term IMT. Three of our patients who were on AZA achieved disease remission, however the number is too small and the studies reviewed did not show convincing outcomes. MTX appears to be the treatment of choice for paediatric patients with VKH.

Finally, Ada appears to show good results. Historically, in the literature to date, most reports have used Ada in VKH only after failed conventional IMT. It would be desirable to have good data from RCTs comparing the use of Ada as a second line agent, after steroids, compared to conventional IMT, and this data is needed as it would be very interesting to see if the use of Ada offered any advantages over conventional IMT in VKH. The early introduction of IMT with Ada and without steroids has already been shown to be safe and effective in a small number of patients and may become a viable treatment option in the future, especially in steroid-intolerant cases [61]. Rituximab may also be a less common option for adalimumab-refractory cases but the data is very limited.

In summary, our series of patients showed historic data on the management trend of VKH, which identified a shift from steroid monotherapy to combined IMT treatment over the last 10 years. This is in agreement with the literature reviewed which showed that early initiation of IMT is essential to achieve immediate disease control and prevent progression to the chronic stage. MMF is the most common IMT choice, which was well tolerated by most patients to achieve disease remission and good visual outcome with minimal VKH-related complications. However, longer term data is required for patients on IMT with larger study size to ascertain and compare overall drug safety profile and its efficacy in preventing chronic-onset VKH and as more recently highlighted, for treatment optimization of subclinical choroidal inflammation [69].

## Data Availability

The datasets generated and analysed is available upon reasonable request and in compliance with local data protection policy.
